# Is There an “Acquired Idiopathic Head-Shaking Nystagmus”?—A Discussion of Mechanisms and Clinical Implications Based on a Case Report

**DOI:** 10.3389/fneur.2022.897012

**Published:** 2022-05-20

**Authors:** Filipp M. Filippopulos, Andreas Zwergal, Doreen Huppert

**Affiliations:** ^1^German Center for Vertigo and Balance Disorders (DSGZ), University Hospital LMU Munich, Munich, Germany; ^2^Department of Neurology, University Hospital Ludwig-Maximilians-Universität (LMU) Munich, Munich, Germany

**Keywords:** head-shaking nystagmus, oculomotor disturbances, vertigo, dizziness, peripheral vestibular system, central vestibular system

## Abstract

**Background:**

Head-shaking nystagmus (HSN) occurs in both peripheral and central vestibular disorders. In most cases, HSN can be attributed to an asymmetric peripheral vestibular input or a structural lesion mostly in the cerebellum affecting the central velocity storage mechanism. An isolated HSN is very rare.

**Case Presentation:**

We report on a young female patient with the clinical picture of recurrent episodes of vertigo, which were induced by fast head movements and were accompanied by a severe right-beating HSN with a long time constant of 60 s. There was no other clinical and instrument-based evidence of peripheral vestibular dysfunction (including video head impulse test, caloric test, vestibular-evoked myogenic potentials) or indication of a structural lesion in the nodulus, uvula or pontomedullary brainstem on fine-slice magnetic resonance imaging. She had no previous history of migraine, hearing deficits, or other focal neurological symptoms. Diagnostic criteria for vestibular paroxysmia, vestibular migraine, benign peripheral paroxysmal vertigo, or any other known vestibular disorders were not fulfilled. Chart review in the database of the German Center for Vertigo and Balance Disorders indicated eight additional patients with a similar clinical phenotype between 2018 and 2022.

**Conclusion:**

We propose a clinical entity called acquired idiopathic head shaking nystagmus (aiHSN) as a rare cause of episodic vertigo induced by fast head movements. Nystagmus characteristics suggest a subtle functional pathology of the central velocity storage mechanism in the nodulus and uvula, which is exacerbated during symptomatic episodes.

## Introduction

Head-shaking nystagmus (HSN) is a distinct ocular motor finding, which is considered to occur in cases with an imbalance in the bilateral peripheral and/or central vestibular system (1, 2). In the case of a reduced unilateral peripheral vestibular input, HSN occurs during head-shaking in accordance with Ewald's second law. HSN is modulated by mechanisms of central velocity storage located mainly in the nodulus and uvula (1, 3). In consequence, lesions of the central vestibular system can also cause HSN, which does not necessarily follow Ewald's second law and appears as so-called perverted HSN (pHSN) (the direction of HSN differs from the direction of the head shaking) (2). The mechanisms leading to pHSN though are not fully understood and lesions to different central structures have been reported to induce pHSN (4–7).

Overall, HSN may occur in a broad spectrum of diseases of the vestibular system including central vestibular disorders (e.g., stroke), vestibular migraine, and diseases mainly affecting the peripheral vestibular organs such as unilateral vestibulopathy or Menière disease (2, 8, 9). Recently, a disorder was characterized by recurrent spontaneous vertigo of unknown etiology and interictal head-shaking nystagmus (RSV-HSN) in the broader spectrum of patients with benign recurrent vertigo (BRV) (10). Patients with RSV-HSN showed recurring episodes of vertigo with no accompanying symptoms indicative of vestibular migraine or Menière's disease and with an interictal presence of HSN. Furthermore, these patients showed no evidence of peripheral or central vestibular deficits. Cases in which HSN occurs as an isolated symptom are extremely rare, but might indicate a more severe underlying cause (6), so that a broad diagnostic approach is merited.

We present a case of a young female patient with isolated recurrent HSN without evidence of any underlying cause after an extensive diagnostic workup including neurologic examination, neuro-orthoptic examination, instrument-based vestibular diagnostics (e.g., video head impulse test, caloric stimulation, vestibular evoked myogenic potentials), and magnetic resonance imaging (MRI). We discuss the potential clinical impact and the diagnostic approach, as well as therapeutic options. Furthermore, we hypothesize the existence of a distinct clinical entity for a subgroup of patients with “acquired idiopathic head-shaking nystagmus”.

## Case Description

A 31-year-old female presented to the German Center for Vertigo and Balance Disorders, (DSGZ) at the University Hospital of the Ludwig-Maximilians-Universität (LMU), Munich, Germany with recurrent episodes of vertigo exclusively trigger by fast head movements. Symptoms initially occurred 2 years prior to the first presentation at the DSGZ during a pregnancy. Since then, she had four episodes lasting for 2–6 weeks each. During these episodes, the patient described vertigo lasting between 20 and 60 s, that was consistently triggered only by head movements. No accompanying symptoms, including symptoms indicative for migraine (e.g., focal neurological symptoms, headache, photo-/phonophobia, aural fullness, hearing loss, tinnitus) were reported. Regarding a possible migraine triggered by hormonal changes during pregnancy, the patient reported no symptoms that might suggest a migrainous predisposition such as motion sickness, reoccurring headaches, or association with menstruation, weather changes, or certain foods. The further patient history and family history, especially regarding migraine, were completely unremarkable. She took no regular medication and had no history of nicotine, drug, or alcohol abuse. Two prior outpatient neurological evaluations during symptomatic episodes suspected unilateral vestibulopathy as an underlying cause, although no peripheral vestibular deficit in the head-impulse test (HIT) or caloric stimulation had been documented.

In the initial clinical evaluation at our center, the patient was awake and fully oriented. The examination of the cranial nerves, motor and sensory function was completely normal. There were no deficits of fine motor skills, nor evidence of ataxia or walking difficulties. Speech and higher cognitive functions were also normal. The ocular motor testing, however, revealed a fast, right-beating HSN with a peak intensity of 13 degrees per second and a frequency of 3 Hz that persisted for up to 60 s after head shaking (see Supplementary Material). The nystagmus was not suppressed by visual fixation and no nystagmus reversal was present at any time-point. The nystagmus was induced by head-shaking with a frequency of at least 2–3 Hz and head excursions of 20–25 deg. Further ocular motor testing, including saccade function and HIT, as well as the Dix-Hallpike maneuver and supine roll test were without pathological findings. No head-bending or lying-down nystagmus was noted. A complete neuro-otological workup including orthoptic evaluation, vestibular hot-/cold-water caloric testing, video-oculography, video HIT, hearing test, posturography, as well as cervical and ocular vestibular evoked myogenic potentials (c/oVEMPs) was conducted. A summary of the findings can be found in Table 1. Besides the aforementioned right-beating HSN, the only pathological finding was a 3-degree deviation of the subjective visual vertical (SVV) to the left.

**Table 1:** Summary of the findings of broad neuro-orthoptic and instrument-based examinations over a follow-up period of 6 years.

**Time point**			**Initial presentation *(within episode)***	**After 1 week *(out of episode)***	**After 4 years *(out of episode)***	**After 5 years *(within episode)***
Neuro-orthoptic examinations	Head-shaking nystagmus	Direction	Right-beating	None	None	Right-beating
		deg/sec	13			14
		Frequency (Hz)	3			3–4
	Positional maneuvers (Dix-Hallpike, supine roll test)		No nystagmus	No nystagmus	No nystagmus	No nystagmus
	Head impulse test right/left		Normal/normal	Normal/normal	Normal/normal	Normal/normal
	Visual acuity right/left		1.0/1.0	1.0/1.0	1.0/1.0	1.0/1.0
	Eye alignment	near	Orthophoria	Orthophoria	Orthophoria	Orthophoria
		distance	Orthophoria	Orthophoria	Orthophoria	Orthophoria
	Convergence		Normal	Normal	Normal	Normal
	Gaze-holding		Normal	Normal	Normal	Normal
	Optokinetic nystagmus		Normal	Normal	Normal	Normal
	Fixation suppression		Normal	Normal-	Normal-	Normal
	Hyperventilation		No nystagmus	No nystagmus	No nystagmus	No nystagmus
	Politzer balloon		No nystagmus	-	-	-
	Smooth pursuit	Horizontal	Smooth	Smooth	Smooth	Smooth
		Vertical	Smooth	Slightly saccadic	Slightly saccadic	Slightly saccadic
	Saccades		Metric	Metric	Metric	Metric
Instrument-based examinations	Video head impulse test gain	Right	0.84	-	0.95	0.88
		Left	0.94	-	0.90	0.91
	Subjective visual vertical deviation (hemispherical dome method)		3° to the left	None	None	None
	Eye fundus (Scanning laser ophthalmoscope)		No torsion	No torsion	No torsion	
	Caloric stimulation (deg/sec)	Left	10.9	-	13.8	14.6
		Right	11.5		15.0	12.4
	Audiometry		Normal hearing	-	Normal hearing	-
	Posturography		Normal sway pattern	-	-	-
	AC-cVEMPs and BC-oVEMPs		Normal	-	-	-

*deg, degrees, sec, seconds, Hz, Hertz, AC-cVEMPs, Air -Conducted cervical Vestibular Evoked Myogenic Potentials, BC-oVEMPs, Bone-Conducted ocular Vestibular Evoked Myogenic Potentials*.

Magnetic resonance imaging (MRI) including 1 mm slices of the brainstem and cerebellum, as well as a CISS (constructive interference in steady state) sequence was normal with no evidence of structural abnormalities of the vestibular organ or evidence of a vascular compression of the 8th cranial nerve. Laboratory workup, including complete blood count, renal and liver function tests, vitamin B1 and B6, as well as basic immunologic workup (antinuclear antibodies, antimitochondrial antibodies, antineutrophil cytoplasmic antibodies, rheumatoid factor, and cardiolipin antibodies), was normal with no evidence of a vitamin deficiency, autoimmune or infectious disease.

In 6 years of follow-up, episodes of HSN occurred every 3–12 months and lasted for 1–2 weeks. Documentation by the patient and the general practitioner revealed a right-beating HSN in the majority of episodes without other accompanying symptoms. Follow-up visits in our center revealed no SVV deviation, peripheral vestibular dysfunction or central neuro-orthoptic deficit, despite a slight vertical saccadic smooth pursuit (Table 1). In the attack-free interval, no HSN, SVV deviation, or other peripheral or central vestibular deficit (despite the above-mentioned vertical saccadic smooth pursuit) was detected. Several therapeutic trials, including physical therapy, exercise, regular magnesium intake (300 mg/d), and transient administration of the sodium channel blocker carbamazepine, had no effect on the recurrence of vertigo episodes.

A revision of cases with HSN at our center revealed eight additional patients since 2018 with very similar symptoms of isolated HSN, which could not be explained by any other peripheral or central vestibular disorder. These patients were 41 ± 14.5 years old. Men and women were equally represented.

## Discussion

We present a rare case of episodically occurring, isolated HSN in a young female patient, followed up over a period of 6 years. Besides the right-beating HSN and a slight transient SVV deviation to the left, no neurological, neuro-otological, or neuro-orthoptic deficits were found. The presentation was not consistent with another vestibular disorders (such as vestibular migraine, or vestibular paroxysmia).

HSN or pHSN are found in different peripheral and central vestibular disorders, but almost never occur without any other ocular motor signs at symptom onset or without any pathological findings in the peripheral or central vestibular system. HSN of suspected peripheral vestibular origin is most commonly present in vestibular neuritis, Menière disease (MD) and benign paroxysmal positional vertigo (especially of the horizontal semicircular canal) (8). Furthermore, HSN has been shown to be present in vestibular paroxysmia (11). In the case of peripheral HSN, the direction of the nystagmus beats in most cases toward the side of the unaffected vestibular organ (monophasic HSN) but may change direction over time (biphasic HSN), or more rarely beats toward the affected side first (reversed biphasic HSN) (8, 12, 13). In central disorders, HSN occurs less frequently and may also manifest as perverted HSN (pHSN) (2). The most frequent central causes of HSN are brainstem or cerebellar infarction and vestibular migraine (2, 14). Regarding cerebral stroke, lesions involving the nodulus, uvula, and inferior tonsil, as well as infarction of the lateral medulla have been shown to cause HSN/pHSN (4, 9). The neural mechanisms underlying HSN in central disorders are widely unknown. One common hypothesis is that the nodulus and uvula play an important role in central vestibular velocity storage that becomes asymmetrical in the case of unilateral infarction of the nodulus/uvula or their projections to the brainstem (i.e., pons and medulla), resulting in ipsilesional HSN (4, 9, 15). pHSN is further explained by a cross-coupling between horizontal and vertical velocity storage pathways (5, 6). In vestibular migraine, where HSN or pHSN can be observed within or between attacks, amplification of a peripheral vestibular asymmetry by the central velocity storage mechanism is assumed (14).

In the present case, a peripheral cause for the episodic HSN can be broadly excluded, especially since in 6 years of follow up no peripheral deficit was ever noted in head impulse or caloric testing. Contrast-enhanced MRI showed no vestibular schwannoma. Furthermore, there was no evidence for a pathological irrigation of peripheral vestibular afferents (e.g., in the context of BPPV or vestibular paroxysmia). The long time constant of the HSN in our case is not compatible with a peripheral HSN type, as previous systematic investigations in patients with unilateral peripheral vestibulopathies revealed a maximum HSN time constant of 20 s (16). A few cases have previously been described with a peripheral syndrome labeled “head-jolting nystagmus”, where rapid head-shaking induced horizontal nystagmus lasting up to 45 s (17, 18). The authors attributed the long-lasting nystagmus to dislodged material within the horizontal semicircular canal. In contrast to the present case the symptoms of these patients could be elicited continuously (not episodically), were suppressed by visual fixation (nystagmus lasted significantly shorter in light vs. dark) and showed evidence of peripheral vestibular dysfunction (e.g., by caloric hypofunction). Thus, “head-jolting nystagmus” seems an unlikely cause in the present case.

Regarding the central vestibular system, an MRI with 1 mm slices of the brainstem and cerebellum did not reveal any structural abnormalities, especially in the nodulus, uvula, medulla, and pons. Accordingly, neuro-orthoptic examination revealed no evidence for a focal central ocular motor disorder indicative of a structural lesion.

In 1979 Slater (19) suggested the term benign recurrent vertigo (BRV) for patients with recurrent vertigo attacks in the absence of any additional otological or neurological symptoms such as hearing loss or headache, and without evidence of peripheral or central vestibular deficits. Since then, there have been attempts to better classify these patients and evaluate possible causes (20–22), but the etiology broadly remains unknown. Most commonly, BRV is thought to be in the spectrum of migraine or MD, although only few patients with BRV have been shown to develop migraine or MD over time (20, 23, 24). One study found HSN in the attack-free period of about 10% of BRV patients and suggested a disorder characterized by recurrent spontaneous vertigo of unknown etiology and interictal head-shaking nystagmus (RSV-HSN) (10). In contrast to the patients described as RSV-HSV, in the present case study the patient only showed HSN during certain, recurring periods and not in the interictal interval. Furthermore, the patient presented here did not exhibit spontaneous vertigo attacks without any apparent trigger such as weather change, menstruation, certain foods, medication. Vertigo, which was only elicited by head-shaking, lasted as long as the HSN persisted, and only occurred in the ictal period. Therefore, we argue that our patient does not entirely fit the concept of BRV or RSV-HSV. To our knowledge, there are no similar cases described in literature. Based on the current case and a cohort of patients with similar clinical presentations, seen in our center, we suggest a distinct disorder characterized by an “acquired idiopathic head-shaking nystagmus” (aiHSN). Our experience shows that patients in the third and fourth decade of life are affected most by aiHSN. Due to the clinical and instrument-based findings, a peripheral cause can widely be excluded. Therefore, based on the nystagmus characteristics (long time constant) and the current literature on HSN/pHSN, we hypothesize that a recurrent functional asymmetry of the central velocity storage might be the most likely cause, despite a missing central structural lesion. The underlying etiology for this is unknown. From a theoretical perspective, the following possibilities may be considered: (1) Temporary decompensation of a slight (undetectable) peripheral vestibular deficit that under normal circumstances is fully centrally compensated. The temporary decompensation might be due to a transient imbalance of neurotransmitters at the level of the nodulus and uvula or vestibular nuclei. Factors leading to such a transient decompensation are only speculative (e.g., hormones). (2) Transient imbalance of the central velocity storage possibly also due to a neurotransmitter imbalance. Mechanisms leading to the imbalance might be related to migraine pathophysiology. (3) An undetectable central microlesion (e.g., at the vestibular nuclei, nodulus, cerebellar peduncles) that might lead to recurrent excitations. Overall, none of these theories can be supported sufficiently with evidence from the here presented cases. Even after extensive and repeated evaluation, there was no evidence of vestibular migraine, a central or peripheral vestibular lesion, or vestibular paroxysmia. From a therapeutic perspective, a specific treatment does not seem to be available. Nevertheless, we suggest habituation and balance exercises to suppress the vertigo sensation induced by HSN.

Future research on patients with aiHSN should focus on experimental testing of the central velocity storage function using established paradigms such as tilt-suppression of the post-rotatory nystagmus (25, 26).

## Data Availability Statement

The raw data supporting the conclusions of this article will be made available by the authors, without undue reservation.

## Ethics Statement

Ethical review and approval was not required for the study on human participants in accordance with the local legislation and institutional requirements. The patients/participants provided their written informed consent to participate in this study. Written informed consent was obtained from the individual(s) for the publication of any potentially identifiable images or data included in this article.

## Author Contributions

FF: patient treatment, acquisition and interpretation of data, and drafting the manuscript. AZ: interpretation of data and revising the manuscript. DH: patient treatment, acquisition and interpretation of data, and drafting the manuscript. All authors approved the final version of the manuscript.

## Conflict of Interest

The authors declare that the research was conducted in the absence of any commercial or financial relationships that could be construed as a potential conflict of interest.

## Publisher's Note

All claims expressed in this article are solely those of the authors and do not necessarily represent those of their affiliated organizations, or those of the publisher, the editors and the reviewers. Any product that may be evaluated in this article, or claim that may be made by its manufacturer, is not guaranteed or endorsed by the publisher.
